# Resource Allocation in Cognitive Radio Wireless Sensor Networks with Energy Harvesting

**DOI:** 10.3390/s19235115

**Published:** 2019-11-22

**Authors:** Haitao Xu, Hongjie Gao, Chengcheng Zhou, Ruifeng Duan, Xianwei Zhou

**Affiliations:** Department of Communication Engineering, University of Science and Technology Beijing, Beijing 10083, China; b20170312@xs.ustb.edu.cn (H.G.); czho9311@163.com (C.Z.); g20188731@xs.ustb.edu.cn (R.D.); xwzhouli@sina.com (X.Z.)

**Keywords:** differential game, resource allocation, open loop Nash equilibrium, feedback Nash Equilibrium

## Abstract

The progress of science and technology and the expansion of the Internet of Things make the information transmission between communication infrastructure and wireless sensors become more and more convenient. For the power-limited wireless sensors, the life time can be extended through the energy-harvesting technique. Additionally, wireless sensors can use the unauthored spectrum resource to complete certain information transmission tasks based on cognitive radio. Harvesting enough energy from the environments, the wireless sensors, works as the second users (SUs) can lease spectrum resource from the primary user (PU) to finish their task and bring additional transmission cost to themselves. To minimize the overall cost of SUs and to maximize the spectrum profit of the PU during the information transmission period, we formulated a differential game model to solve the resource allocation problem in the cognitive radio wireless sensor networks with energy harvesting, considering the SUs as the game players. By solving the proposed resource allocation game model, we found the open loop Nash equilibrium solutions and feedback Nash equilibrium solutions for all SUs as the optimal control strategies. Ultimately, series numerical simulation experiments have been made to demonstrate the rationality and effectiveness of the game model.

## 1. Introduction

With the development of the Internet of Things (IoTs), all things will have the ability to perceive the environment. Through the deep integration of the physical world and the digital world [1], the IoTs revolutionizes connection and interaction among all objects. Based on the IoT, the physical equipment and physical world are no longer cold but become a vitality community. In addition, wireless sensor networks (WSN), as the nerve ending of IoTs, have attracted extensive attention and research interest by many scholars in recent years [2], and their theoretical and applied research has received more and more attention [3].

WSNs are composed of a series of sensor nodes. Generally, communication between the sensor nodes is completed using the common frequency band. However, with the development of wireless communication, this frequency band is increasingly crowded, and the communication between sensor nodes receives interference not only by their own nodes, but also increasingly serious and uncontrollable interference from other application type networks. As interconnected devices increase rapidly, sensor nodes with multiple applications overlapping coverage areas will suffer from more severe interference. Over the past few years, cognitive radio networks have been proposed as an effective way to alleviate the spectrum scarcity problem [4]. Many scholars have realized that cognitive radio technology can be an effective means to improve the utilization of spectrum resources and have done a great deal of research on it [5].

By configuring the cognitive radio module to the sensor node, we can detect the state of the licensed spectrum and opportunistically utilize the idle licensed spectrum for data transmission [6]. A wireless network of sensor nodes with cognitive radio modules is also known as a cognitive radio wireless sensor network (CRWSN), which is no longer subject to signal interference from the public frequency band. Although CRWSNs are no longer subject to transmission interference caused by a lack of spectrum, nodes in the sensor network need to consume additional energy to implement cognitive radio functions, such as spectrum detection, spectrum switching, etc., which are generally powered by difficult-to-replace batteries. For sensor nodes, the problem of insufficient energy becomes more severe. Therefore, compared with traditional wireless sensor networks, the network lifetime problem of CRWSNs caused by insufficient node energy becomes more urgent [7]. In order to overcome the lack of nodes and ensure that the network can continue to operate effectively, energy-harvesting technology has begun to be adopted by sensor nodes. Using energy-harvesting technology, sensor nodes can collect ambient conditions such as solar energy, wind energy, and vibration energy [8,9]. The nodes continuously obtain energy from the surrounding environment, and the lifetime of the sensor network can be effectively extended [10].

Cognitive radio wireless sensor networks with energy harvesting can achieve a continuous supply of spectrum resources and energy resources to make up for the shortcomings of traditional sensor networks [11]. Compared with traditional sensor networks, CRWSNs with energy harvesting have obvious advantages and great development potentials, which brings new opportunities to the development of sensor networks and lays a foundation for the development of the Internet of Things. However, both energy and spectrum resources in the CRWSNs are dynamic and difficult to predict. The sensor node performs spectrum detection by consuming energy to obtain transmission opportunities. Under the guarantee of sufficient residual energy, the sensor node can use this opportunity to transmit the collected data; otherwise, even if there is a transmission opportunity, the sensor node will not have enough energy and no data transmission or data collection. 

Therefore, the resource allocation of CRWSNs with energy harvesting needs to consider the dynamic energy-harvesting process and the random available spectrum resources. Researchers have made some achievements in the research of the above problems. Firstly, there are three popular resource allocation schemes [12] in the field of cognitive radio sensor networks: centralized, cluster-based, and distributed scenarios. The centralized scheme means that the central node of the cognitive radio network is the network backbone, which can control the power of other sensing nodes in the network and make decisions on resource allocation. The centralized scheme can be found in the literature [13,14,15,16], which achieves multiple optimization goals through efficient decision making, such as improving energy efficiency and maximizing throughput. However, the control channel transmits information that requires higher power, and the network is susceptible to central node failure. The cluster-based scheme means that the network is divided into multiple clusters, and each cluster has one cluster head and multiple intracluster nodes. After the cluster head aggregates the information, it makes the decision of resource allocation and communicates to each node. A number of cluster-based approaches have been adopted in some of the literature [17,18,19,20]. There are fewer members in the cluster, which can reduce the signaling overhead for each cluster head, but if the cluster head node fails, the connections to neighboring nodes in the cluster may be invalid. The distributed scenarios means that each node in a distributed system makes decisions either autonomously or in cooperation with neighboring nodes. In a time-varying environment, it is possible to quickly adapt to node failures and ensure robustness. However, this solution is based on local information decisions, which are susceptible to interference and malicious attacks, resulting in inefficient solutions [21,22,23].

Additionally, lots of productive work has been done on the resource allocation problem under the cognitive radio networks with energy harvesting. Previous work focused mainly on the optimal spectrum sensing algorithms to improve spectrum utilization efficiency [24]. There are also some studies that focused on transmission strategies, which aim to improve energy use efficiency and choose different channel models to improve the efficiency of energy collections [25,26]. Recently, cognitive radio network energy-harvesting systems based on spectrum sharing have become a significant research direction. A novel energy cooperation transmission scheme was proposed for cognitive spectrum-sharing-based D2D communication system in [27]. Li et al. [28] considered a spectrum-sharing scheme based on simultaneous wireless information and power transfer (SWIPT). Zhang et al. [29] proposed a new cooperative spectrum-sharing protocol with dynamic time-slot allocation based on energy harvesting. However, none of the related works mentioned above takes into account resource allocation in CRWSNs with energy harvesting.

In CRWSNs with energy harvesting, the wireless sensors can be considered as the secondary users (SUs), and the base station or infrastructure can be seen as the primary user (PU). The SUs can capture the spectrum from the PU. Before information transmission, the SUs should harvest enough energy for information transmission. Then, when the channels are not occupied by the PU, the SUs will use the harvest energy to transmit information. The available information transmission spectrum mainly depends on the spectrum leased from the PU. The SUs should control their spectrum requirements to lower the spectrum lease cost, while completing the information transmission tasks. In this paper, we investigated the optimal resource allocation problem for the PUs and SU based on a differential game. The SUs control their spectrum requirements to minimize the cost. 

In this paper, we propose a differential game-based resource allocation problem in CRWSN. The system state of the proposed CRWSN is the capacity of the spectrum resource that the PU wants to lease. All the wireless sensors that can be seen as SUs should control the resource level leased from the PU to minimize their cost during the information transmission. We obtained open loop and feedback Nash equilibria from the SUs. The numerical results are given to present the correctness of the differential game analysis. The whole paper is organized as follows: Section 2 is the system differential game model and problem formulation, which consists of two parts, that is, system model and game formulation. Section 3 is the game analysis, which consists of three parts, that is, open loop Nash equilibrium, feedback Nash equilibrium under the finite horizon, and feedback Nash equilibrium under the infinite horizon. Section 4 is a numerical simulation and analysis. Finally, there is the conclusion about the main work and the corresponding summary in Section 5.

## 2. System Model and Problem Formulation

### 2.1. System Model

**Figure 1:**
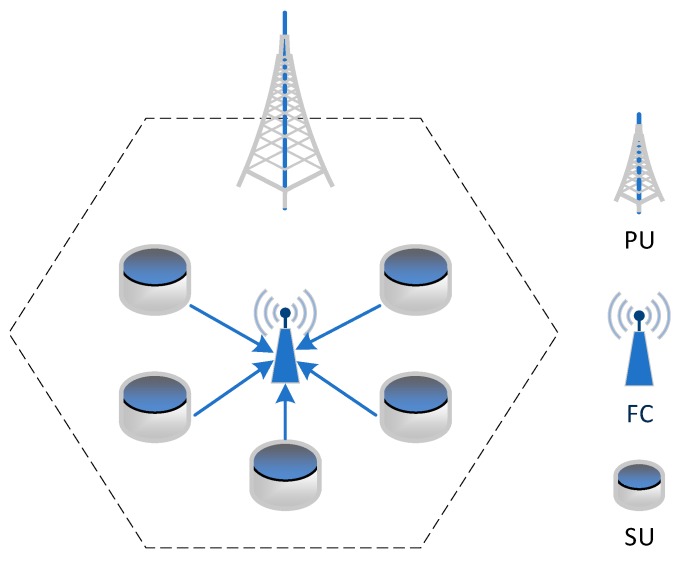
Cognitive radio wireless sensor network (CRWSN) system model.

As shown in Figure 1, here, we consider a CRWSN with one primary user (PU), one fusion center (FC), and N secondary users (SUs) with N≥2. All the SUs in the SU system share the same spectrum band with the PU. Meanwhile, the SUs communicates with the FC via the same channel of the PU. We assume that the SU is equipped with the energy harvest circuits. The SUs can harvest energy from the ambient environment (e.g., solar, wind, and radio frequency) to increase the lifetime. The harvest energy is stored in a rechargeable battery with finite energy storage capacity. With the harvest energy, the SUs can achieve information transmission with the FC. The SUs can harvest energy when the PU’s spectrum is busy. The status of the spectrum is updated by the PU. When the spectrum is not used by the PU, the PU will announce its status and lease its spectrum to the SUs. The spectrum status can be seen by the SUs through the available spectrum idle status map [30], which can be dynamically updated by the PU through collaboration with SUs. Using the leased spectrum, the SUs can directly transmit data to the FC with the harvest energy. The work mode of SUs can be considered as the harvest-then-transmit mode.

**Figure 2:**
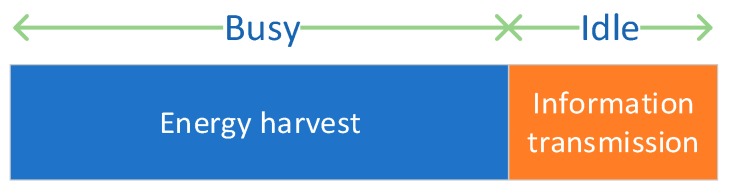
Harvest-then-transmit mode.

Let $u_{i}\left(t\right)$ denote the leased spectrum of SU i at time t, for i∈N, where each SU controls its spectrum band from the PU. Let $U^{i}$ be the set of admissible spectrum band. The harvest-then-transmit mode in our proposed model includes two phases as shown in Figure 2, the energy-harvesting phase and the information transmission phase, respectively. In the energy-harvesting phase, the SUs will harvest enough energy for information transmission. In the information transmission phase, the SUs will use the harvested energy and leased spectrum to complete the information transmission tasks. Let $\beta_{i}$ denote the normalized channel idle period, and $\left(1-\beta_{i}\right)$ is the normalized channel busy period. When the channel is idle, $\beta_{i}$ is the time fraction of the SUs for information transmission. When the channel is busy, $\left(1-\beta_{i}\right)$ is the time fraction of the SUs for energy harvesting. The harvest energy during the $\left(1-\beta_{i}\right)$ period will be stored in the battery of the SUs for information transmission during the $\beta_{i}$ period. For example, the normalized idle period for the Disney TV channel is approximately 0.25 (25%), which means $\beta_{i}=0.25$ for the SUs in the Disney TV channel. Let $\pi_{p}$ denote the spectrum price of the PU when leasing the spectrum to SUs. The PU can adjust its price continuously as a seller during the idle period to maximize the spectrum income. The SUs should adjust the corresponding leased spectrum according to the needs and the spectrum price announced by the PU.

### 2.2. Game Formulation

We formulate the resource allocation problem in the CRWSN with energy harvesting as a differential game as follows:

- *Players*: The set of N SUs in the proposed CRWSN are the players of the differential game;
- *State*: The system state of the proposed CRWSN is the capacity of the spectrum resource the PU wants to lease;
- *Strategy*: The strategy of each SU is the leased spectrum resource from the PU. Accordingly, the strategy set can be denoted as $U^{i}=\left\{u_{1},u_{2},\ldots ,u_{N}\right\}$.

We aim at minimizing the overall cost of the SUs during the energy-harvesting and information transmission period. The overall cost function for each SU is given by the following equation:

(1) $$U_{i}\left(t\right)=\omega_{i}^{sp}U_{i}^{sp}\left(t\right)+\omega_{i}^{eh}U_{i}^{eh}\left(t\right)+\omega_{i}^{dis}U_{i}^{dis}\left(t\right),$$

where $U_{i}^{sp}\left(t\right)$ is the spectrum cost for the spectrum leased from the PU, which is a linear function of the spectrum leased from the PU and is given by $\beta_{i}\pi_{p}u_{i}\left(t\right)$. If the spectrum price $\pi_{p}$ is announced by the PU, the SUs should pay for the leased spectrum to the PU with payment $\beta_{i}\pi_{p}u_{i}\left(t\right)$. Because the battery capacity of the SUs is limited, each SU is equipped with the energy-harvesting circuit to harvest enough energy before the information transmission. $U_{i}^{eh}\left(t\right)$ is the cost of energy harvesting of SU i to have enough energy for information transmission and can be given by $\left(1-\beta_{i}\right)\varepsilon_{i}^{2}u_{i}^{2}\left(t\right)/\eta_{i}$, where $\eta_{i}$ is the conversion efficiency of the harvest energy and $\varepsilon_{i}$ is the QoS requirements of SU i, such as energy efficiency, or spectrum efficiency. Based on the harvest-then-transmit mode, there are two costs during the harvest-then-transmit process, the spectrum cost $U_{i}^{sp}\left(t\right)$ and the energy-harvesting cost $U_{i}^{eh}\left(t\right)$. $U_{i}^{dis}\left(t\right)$ is the discrepancy cost between the spectrum requirements and available capacity of the spectrum resource. Without loss of generality, we use the quadratic function to express the discrepancy cost between the spectrum requirements and the available capacity, and the cost is denoted by ${\left(u_{i}\left(t\right)-x\left(t\right)\right)}^{2}$. $\omega_{i}^{sp}$, $\omega_{i}^{eh}$, and $\omega_{i}^{dis}$ are weighted parameters. In the above equation, we find that we do not consider the interference cost, because we have the spectrum cost due to the spectrum leased from the PU.

The SUs will acquire the availability of spectrum resource at the beginning of the idle time slot from the PU and will share the spectrum resource with the other SUs. To avoid interference among SUs, each of the spectrum resources can only be allocated to one SU. Meanwhile, at the beginning of the idle time slot, the PU will announce a unit price for the leased spectrum. Then, the SUs should decide the spectrum requirements based on the price and their acquirements. Let x(t) be the capacity of the spectrum resource at time t. In particular, we have $U^{i}\in R^{+}$, x(t)>0, and $U^{i}=0$ for x(t)=0. The evolution of spectrum capacity x(t) can be given by the following differential equation:

(2) $$dx\left(t\right)=\left[\sum_{i=1}^{N}\alpha_{i}u_{i}\left(t\right)+\delta x\left(t\right)\right]dt,$$

where $\alpha_{i}$ is a negative constant which means the spectrum leasing efficiency of SU i from the PU. δ is the spectrum loss rate during the spectrum leasing. From Equation (2), we can find that the dynamic variation of the spectrum capacity is mainly controlled by the control variables $u_{i}\left(t\right)$. The evolution of spectrum capacity is an ordinary differential equation of variables $u_{i}\left(t\right)$.

Based on the above models, we formulate the optimal resource allocation problem in the proposed CRWSN. Assuming the observing time for the proposed game is $\left[t_{0},T\right]$, the objective for SU i can be written as differential game as follows:

(3) $$J_{i}\left(t\right)=\underset{u_{i}\left(t\right)}{\min}\left\{\int_{t_{0}}^{T}U_{i}\left(t\right)e^{-rt}dt+\Phi \left(x_{T}\right)\right\}\;=\underset{u_{i}\left(t\right)}{\min}\left\{\int_{t_{0}}^{T}\left[\omega_{i}^{sp}U_{i}^{sp}\left(t\right)+\omega_{i}^{eh}U_{i}^{eh}\left(t\right)+\omega_{i}^{dis}U_{i}^{dis}\left(t\right)\right]e^{-rt}dt+\Phi_{i}\left(x\left(T\right)\right)\right\},$$

subject to:

(4) $$\frac{dx\left(t\right)}{dt}=\sum_{i=1}^{N}\alpha_{i}u_{i}\left(t\right)+\delta x\left(t\right),$$

where $\Phi_{i}\left(x\left(T\right)\right)$ is the terminal cost after the game period that the SUs can achieve, which is given by the spectrum capacity x(t) at the end of the time interval. r is the discount rate and $e^{-rt}$ is the discount factor. SU i is to find the optimal spectrum strategy that can minimize its overall cost function over the time interval $\left[t_{0},T\right]$. In the following section, the equilibrium to the differential game is given based on the Hamilton-Jacob-Bellman (HJB) function.

## 3. Game Analysis

In this section, we try to find the equilibrium solution to the proposed differential game. Based on the spectrum price controlled by the PU, the SUs should control their spectrum requirements to minimize the overall cost given by Equations (3) and (4). Firstly, assuming the initial state of the spectrum capacity is known by all the SUs, we can find the open loop Nash equilibrium for each SU. Then, the feedback solutions to the proposed game are discussed based on Bellman dynamic programming, when the game players know the exact system state at time instant t.

### 3.1. Open Loop Nash Equilibrium

First, we discuss the open loop solutions. In order to get the open loop equilibrium solutions, some definitions are needed as follows.

**Definition** **1.** The allocated spectrum resource $u_{i}^{*}\left(t\right)$ from PU to SU is optimal under the open loop condition if the following inequality holds for all control variables $u_{i}\left(t\right)\;\neq u_{i}^{*}\left(t\right)$ in the set of admissible spectrum $U^{i}$,

(5) $$J_{i}\left(t,u_{i}^{*}\left(t\right)\right)\leq J_{i}\left(t,u_{i}\left(t\right)\right).$$

**Definition** **2.** *The Hamiltonian function of each SU in the proposed differential game in the time period $\left[t_{0},T\right]$ can be given as follows:*

(6) $$H_{i}\left(t,x\right)=U_{i}\left(t\right)e^{-rt}+\Lambda_{i}\left(t\right)\frac{dx}{dt},$$

*where $\Lambda_{i}\left(t\right)$ is the costate function and is given by:*

(7) $$\frac{{d\Lambda }_{i}\left(t\right)}{dt}=-\frac{\partial H_{i}\left(t,x\right)}{\partial x}.$$

**Theorem** **1.** The allocated spectrum resource $u_{i}^{*}\left(t\right)$ provides an open loop Nash equilibrium to the proposed resource allocation game in Equations (3) and (4) if there are constate functions $\Lambda_{i}\left(t\right)$, satisfying the following equations:

(8) $$u_{i}^{*}\left(t\right)=arg\underset{u_{i}\left(t\right)\in U^{i}}{\min}H_{i}\left(t,x\right)=\;arg\underset{u_{i}\left(t\right)\in U^{i}}{\min}\left[U_{i}\left(t\right)e^{-rt}+\Lambda_{i}\left(t\right)\frac{dx}{dt}\right]$$

*Considering the optimal control problem given by Equations (3) and (4), based on the Pontryagin’s maximum principle, we can have the Nash equilibrium solutions of the optimal resource allocation problem for each SU.*

**Theorem** **2.** *The optimal resource allocation strategy of SU i is given by:*

(9) $$u_{i}^{*}\left(t\right)=-{\left(2\omega_{i}^{dis}+2\omega_{i}^{eh}\left(1-\beta_{i}\right)\varepsilon_{i}^{2}\eta_{i}^{-1}\right)}^{-1}\left(\Lambda_{i}e^{rt}\alpha_{i}+\omega_{i}^{sp}\beta_{i}\pi_{p}-2\omega_{i}^{dis}x\left(t\right)\right),$$

*where $\left[x\left(t\right),\Lambda_{i}\left(t\right)\right]$ are the solutions to the following Riccati function:*

(10) $$\frac{{d\Lambda }_{i}\left(t\right)}{dt}=2\omega_{i}^{dis}\left[u_{i}^{*}\left(t\right)-x\left(t\right)\right]e^{-rt}-\Lambda_{i}\left(t\right)\delta ,$$

(11) $$\frac{dx\left(t\right)}{dt}=\sum_{i=1}^{N}\alpha_{i}u_{i}^{*}\left(t\right)+\delta x\left(t\right),$$

*In Equation (9), the allocated spectrum resource for SU i is a linear function of the system state x(t), and affected by the unit spectrum price $\pi_{p}$ controlled by the PU. The SU should make decision for the spectrum from the PU based on the available system capacity x(t) at time t and should consider the influence of unit resource price $\pi_{p}$.*

**Proof.**  For the open loop equilibrium, Pontryagin’s maximum principle can be used as the necessary condition to find the optimal strategies. The Hamiltonian function of the SU is given by Equation (6), and take the derivative of the Hamiltonian function yields:

(12) $$u_{i}^{*}\left(t\right)=-{\left(2\omega_{i}^{dis}+2\omega_{i}^{eh}\left(1-\beta_{i}\right)\varepsilon_{i}^{2}\eta_{i}^{-1}\right)}^{-1}\left(\alpha_{i}\Lambda_{i}e^{rt}+\omega_{i}^{sp}\beta_{i}\pi_{p}-2\omega_{i}^{dis}x\left(t\right)\right),$$

which is the optimal strategy for the SUs under the open loop condition. Based on Equation (12), we can allocate the spectrum resource to SUs for the proposed game durations $\left[t_{0},T\right]$.  □

### 3.2. Feedback Nash Equilibrium under Finite Horizon

In the open loop Nash equilibrium, the system state is not known by all the game players. The game players only know the initial system state. The SUs make decisions on the optimal allocated resource based on the time instant s and the initial system state x(0). Next, we try to find the feedback strategies when the system state is known by the SUs. The optimal solutions to the proposed game under feedback condition depend on the current time and current system state. In order to have the feedback solutions, some definitions are needed.

**Definition** **3.** The allocated spectrum resource $u_{i}^{*}\left(t,x\right)$ from PU to SU is optimal under feedback condition if the following inequality holds for all control variables $u_{i}\left(t,x\right)\;\neq u_{i}^{*}\left(t,x\right)$ in the set of admissible spectrum $U^{i}$:

(13) $$J_{i}\left(t,u_{i}^{*}\left(t,x\right)\right)\leq J_{i}\left(t,u_{i}\left(t,x\right)\right).$$

**Theorem** **3.** The allocated spectrum resource $u_{i}^{*}\left(t,x\right)$ provides a feedback Nash equilibrium to the proposed resource allocation game in Equations (3) and (4) if there are continuously differentiable functions $V_{i}\left(t,x\right)$, satisfying the following set of partial differential equations:

(14) $$-\frac{dV_{i}\left(t,x\right)}{dt}=\underset{u_{i}\left(t,x\right)\in U^{i}}{\min}\left\{U_{i}\left(t,x\right)e^{-rt}+\frac{dV_{i}\left(t,x\right)}{dx}\frac{dx}{dt}\right\},$$

(15) $$V_{i}\left(T,x\left(T\right)\right)=\Phi_{i}\left(x\left(T\right)\right),$$

*where $V_{i}\left(t,x\right)$ is the game equilibrium payoff of SU i at time $t\in \left[t_{0},T\right]$ with the system state being x, which is called value function of SU i in the proposed resource allocation problem.*

**Definition** **4.** *The value function of each SU under feedback control can be given as follows:*

(16) $$V_{i}\left(t,x\right)=\underset{u_{i}}{\min}\left\{\int_{t}^{T}\left[\omega_{i}^{sp}U_{i}^{sp}\left(t\right)+\omega_{i}^{eh}U_{i}^{eh}\left(t\right)+\omega_{i}^{dis}U_{i}^{dis}\left(t\right)\right]e^{-rs}ds+\Phi_{i}\left(x\left(T\right)\right)\right\}\;=\int_{t}^{T}[\omega_{i}^{sp}U_{i}^{sp}\left(s,u_{i}^{*}\left(s,x\right),x^{*}\left(s\right)\right)+\omega_{i}^{eh}U_{i}^{eh}\left(s,u_{i}^{*}\left(s,x\right),x^{*}\left(s\right)\right)+\;\omega_{i}^{dis}U_{i}^{dis}\left(t,u_{i}^{*}\left(s,x\right),x^{*}\left(s\right)\right)]e^{-rs}dt+\Phi_{i}\left(x\left(T\right)\right),$$

*and satisfying the boundary condition:*

(17) $$V_{i}\left(T,x\left(T\right)\right)=\Phi_{i}\left(x\left(T\right)\right).$$

Given another resource allocation strategy $u_{i}\neq u_{i}^{*}$, with the corresponding system state x, then we can have the following equations:

$$U_{i}\left(t,u_{i},x\right)e^{-rt}+\frac{dV_{i}\left(t,u_{i},x\right)}{dx}\frac{dx}{dt}+\frac{dV_{i}\left(t,u_{i},x\right)}{dt}\geq 0,$$

*and:*

$$U_{i}\left(t,u_{i}^{*},x^{*}\right)e^{-rt}+\frac{dV_{i}\left(t,u_{i}^{*},x^{*}\right)}{dx}\frac{dx}{dt}+\frac{dV_{i}\left(t,u_{i}^{*},x^{*}\right)}{dt}=0.$$

*Integrating the above expressions from $t_{0}$ to T, we obtain:*

$$\int_{t_{0}}^{T}U_{i}\left(s,u_{i},x\right)e^{-rs}ds+V\left(T,x\left(T\right)\right)-V\left(t_{0},x_{0}\right)\geq 0,\;\mathrm{and}\;\int_{t_{0}}^{T}U_{i}\left(s,u_{i}^{*},x^{*}\right)e^{-rs}ds+V\left(T,x^{*}\left(T\right)\right)-V\left(t_{0},x_{0}\right)=0.$$

*Performing the indicated minimization in Equations (14) and (15) yields:*

(18) $$u_{i}^{*}\left(t\right)=-{\left(2\omega_{i}^{dis}+2\omega_{i}^{eh}\left(1-\beta_{i}\right)\varepsilon_{i}^{2}\eta_{i}^{-1}\right)}^{-1}\left(\alpha_{i}\frac{dV_{i}\left(t,x\right)}{dx}e^{rt}+\omega_{i}^{sp}\beta_{i}\pi_{p}-2\omega_{i}^{dis}x\left(t\right)\right).$$

*Substituting Equation (18) into Equations (16) and (17) and solving Equations (16) and (17), one obtains:*

(19) $$V_{i}\left(t,x\right)=e^{-rt}\left(\frac{1}{2}A_{i}\left(t\right)x^{2}+B_{i}\left(t\right)x+C_{i}\left(t\right)\right),$$

*where $A_{i}\left(t\right)$, $B_{i}\left(t\right)$ and $C_{i}\left(t\right)$ satisfy the following differential equations:*

(20) $$-\frac{1}{2}\frac{dA_{i}\left(t\right)}{dt}+\frac{1}{2}rA_{i}\left(t\right)={\left(\omega_{i}^{dis}+\omega_{i}^{eh}\left(1-\beta_{i}\right)\varepsilon_{i}^{2}\eta_{i}^{-1}\right)}^{-1}\left(\alpha_{i}A_{i}\left(t\right)-2\omega_{i}^{dis}\right)\;\left(\frac{3}{2}\omega_{i}^{dis}-\frac{1}{4}\alpha_{i}A_{i}\left(t\right)\right)+\omega_{i}^{dis}+\delta A_{i}\left(t\right)\;+A_{i}\left(t\right)\sum_{j=1}^{N}\alpha_{j}{\left(-2\omega_{j}^{dis}-2\omega_{j}^{eh}\left(1-\beta_{j}\right)\varepsilon_{j}^{2}\eta_{j}^{-1}\right)}^{-1}\left(\alpha_{j}A_{j}\left(t\right)-2\omega_{j}^{dis}\right)$$

(21) $$-\frac{dB_{i}\left(t\right)}{dt}+rB_{i}\left(t\right)=\delta B_{i}\left(t\right)-\frac{1}{2}\omega_{i}^{sp}\beta_{i}\pi_{p}{\left(\omega_{i}^{dis}+\omega_{i}^{eh}\left(1-\beta_{i}\right)\varepsilon_{i}^{2}\eta_{i}^{-1}\right)}^{-1}\left(\alpha_{i}A_{i}\left(t\right)-2\omega_{i}^{dis}\right)\;+\frac{1}{2}\alpha_{i}A_{i}\left(t\right){\left(\omega_{i}^{dis}+\omega_{i}^{eh}\left(1-\beta_{i}\right)\varepsilon_{i}^{2}\eta_{i}^{-1}\right)}^{-1}\left(\alpha_{i}B_{i}\left(t\right)+\omega_{i}^{sp}\beta_{i}\pi_{p}\right)\;+A_{i}\left(t\right)\sum_{j=1}^{N}\alpha_{j}{\left(-2\omega_{j}^{dis}-2\omega_{j}^{eh}\left(1-\beta_{j}\right)\varepsilon_{j}^{2}\eta_{j}^{-1}\right)}^{-1}\left(\alpha_{j}B_{j}\left(t\right)+\omega_{j}^{sp}\beta_{j}\pi_{p}\right)\;+B_{i}\left(t\right)\sum_{j=1}^{N}\alpha_{j}{\left(-2\omega_{j}^{dis}-2\omega_{j}^{eh}\left(1-\beta_{j}\right)\varepsilon_{j}^{2}\eta_{j}^{-1}\right)}^{-1}\left(\alpha_{j}A_{j}\left(t\right)-2\omega_{j}^{dis}\right).$$

(22) $$-\frac{dC_{i}\left(t\right)}{dt}+rC_{i}\left(t\right)=\frac{1}{4}{\left(\omega_{i}^{dis}+\omega_{i}^{eh}\left(1-\beta_{i}\right)\varepsilon_{i}^{2}\eta_{i}^{-1}\right)}^{-1}\left({\left(\alpha_{i}B_{i}\left(t\right)\right)}^{2}-{\left(\omega_{i}^{sp}\beta_{i}\pi_{p}\right)}^{2}\right)\;+B_{i}\left(t\right)\sum_{j=1}^{N}\alpha_{j}{\left(-2\omega_{i}^{dis}-2\omega_{i}^{eh}\left(1-\beta_{i}\right)\varepsilon_{i}^{2}\eta_{i}^{-1}\right)}^{-1}\left(\alpha_{j}B_{j}\left(t\right)+\omega_{i}^{sp}\beta_{i}\pi_{p}\right).$$

**Theorem** **4.** *The optimal resource allocation strategy of SU i under the feedback control situation is given by:*

(23) $$u_{i}^{*}\left(t\right)=-{\left(2\omega_{i}^{dis}+2\omega_{i}^{eh}\left(1-\beta_{i}\right)\varepsilon_{i}^{2}\eta_{i}^{-1}\right)}^{-1}\left(\alpha_{i}\left(A_{i}\left(t\right)x+B_{i}\left(t\right)\right)+\omega_{i}^{sp}\beta_{i}\pi_{p}-2\omega_{i}^{dis}x\left(t\right)\right),$$

*where $A_{i}\left(t\right)$ and $B_{i}\left(t\right)$ are the solutions of the differential equations given by Equations (20)–(22).*

### 3.3. Feedback Nash Equilibrium under Infinite Horizon

We now turn the proposed game to the infinite-horizon autonomous game with a constant discount factor. In this subsection, the observing time for the differential game is infinity. The objective function and system state function, which are given by Equations (3) and (4), are both non-autonomous. The game problem in Equations (3) and (4) is changed to a problem with infinite horizon as follows:

(24) $$J_{i}\left(t\right)=\underset{u_{i}\left(t\right)}{\min}\left\{\int_{t_{0}}^{\infty }U_{i}\left(t\right)e^{-rt}dt\right\}\;=\underset{u_{i}\left(t\right)}{\min}\left\{\int_{t_{0}}^{\infty }\left[\omega_{i}^{sp}U_{i}^{sp}\left(t\right)+\omega_{i}^{eh}U_{i}^{eh}\left(t\right)+\omega_{i}^{dis}U_{i}^{dis}\left(t\right)\right]e^{-rt}dt\right\},$$

subject to:

(25) $$dx\left(t\right)=\left[\sum_{i=1}^{N}\alpha_{i}u_{i}\left(t\right)+\delta x\left(t\right)\right]dt.$$

Under the infinite horizon, the solutions are independent of time-instant and dependent only on the system state at the starting time. A feedback solution for the infinite horizon game in Equations (24) and (25) can be characterized as follows.

**Theorem** **5.** The allocated spectrum resource $u_{i}^{*}\left(t,x\right)$ provides a feedback Nash equilibrium to the proposed resource allocation game in Equations (24) and (25) if there are functions $W_{i}\left(t,x\right)$, satisfying the following set of partial differential equations:

(26) $$rW_{i}\left(x\right)=\underset{u_{i}\left(x\right)\in U^{i}}{\min}\left\{\omega_{i}^{sp}U_{i}^{sp}+\omega_{i}^{eh}U_{i}^{eh}+\omega_{i}^{dis}U_{i}^{dis}+\frac{dW_{i}\left(x\right)}{dx}\frac{dx}{dt}\right\}.$$

**Theorem** **6.** *The optimal resource allocation strategy of SU i is given by:*

(27) $$u_{i}^{*}=-{\left(2\omega_{i}^{dis}+2\omega_{i}^{eh}\left(1-\beta_{i}\right)\varepsilon_{i}^{2}\eta_{i}^{-1}\right)}^{-1}\left(\frac{dW_{i}\left(x\right)}{dx}\alpha_{i}+\omega_{i}^{sp}\beta_{i}\pi_{p}-2\omega_{i}^{dis}x\right),$$

**Proof.**  Performing the indicated minimization in Equation (27), we can obtain:

(28) $$u_{i}^{*}=-{\left(2\omega_{i}^{dis}+2\omega_{i}^{eh}\left(1-\beta_{i}\right)\varepsilon_{i}^{2}\eta_{i}^{-1}\right)}^{-1}\left(\frac{dW_{i}\left(x\right)}{dx}\alpha_{i}+\omega_{i}^{sp}\beta_{i}\pi_{p}-2\omega_{i}^{dis}x\right).$$

Incorporating the solution $u_{i}^{*}$ into Equations (24) and (25), and solving the equations, we can obtain:

(29) $$W_{i}\left(x\right)=\frac{1}{2}rWA_{i}\left(x\right)x^{2}+rWB_{i}\left(x\right)x+rWC_{i}\left(x\right),$$

where $WA_{i}\left(x\right)$, $WB_{i}\left(x\right),$ and $WC_{i}\left(x\right)$ satisfy the following equations:

(30) $$\frac{1}{2}rWA_{i}\left(x\right)=\omega_{i}^{dis}+\delta WA_{i}\left(x\right)+\frac{1}{4}{\left(\omega_{i}^{dis}+\omega_{i}^{eh}\left(1-\beta_{i}\right)\varepsilon_{i}^{2}\eta_{i}^{-1}\right)}^{-1}\left({\left(\alpha_{i}WA_{i}\left(x\right)\right)}^{2}-{\left(2\omega_{i}^{dis}\right)}^{2}\right)\;+WA_{i}\left(x\right)\sum_{j=1}^{N}\alpha_{j}{\left(-2\omega_{j}^{dis}-2\omega_{j}^{eh}\left(1-\beta_{j}\right)\varepsilon_{j}^{2}\eta_{j}^{-1}\right)}^{-1}\left(\alpha_{j}WA_{j}\left(x\right)-2\omega_{j}^{dis}\right)$$

(31) $$rWB_{i}\left(x\right)=\delta WB_{i}\left(x\right)+\frac{1}{2}\alpha_{i}WB_{i}\left(x\right){\left(\omega_{i}^{dis}+\omega_{i}^{eh}\left(1-\beta_{i}\right)\varepsilon_{i}^{2}\eta_{i}^{-1}\right)}^{-1}\left(\alpha_{i}WA_{i}\left(x\right)-2\omega_{i}^{dis}\right)\;+\omega_{i}^{dis}{\left(\omega_{i}^{dis}+\omega_{i}^{eh}\left(1-\beta_{i}\right)\varepsilon_{i}^{2}\eta_{i}^{-1}\right)}^{-1}\left(\alpha_{i}WB_{i}\left(x\right)+\omega_{i}^{sp}\beta_{i}\pi_{p}\right)\;+WA_{i}\left(x\right)\sum_{j=1}^{N}\alpha_{j}{\left(-2\omega_{j}^{dis}-2\omega_{j}^{eh}\left(1-\beta_{j}\right)\varepsilon_{j}^{2}\eta_{j}^{-1}\right)}^{-1}\left(\alpha_{j}WB_{j}\left(x\right)+\omega_{j}^{sp}\beta_{j}\pi_{p}\right)\;+WB_{i}\left(x\right)\sum_{j=1}^{N}\alpha_{j}{\left(-2\omega_{i}^{dis}-2\omega_{i}^{eh}\left(1-\beta_{i}\right)\varepsilon_{i}^{2}\eta_{i}^{-1}\right)}^{-1}\left(\alpha_{j}WA_{j}\left(x\right)-2\omega_{j}^{dis}\right)$$

(32) $$rWC_{i}\left(x\right)=\frac{1}{4}{\left(\omega_{i}^{dis}+\omega_{i}^{eh}\left(1-\beta_{i}\right)\varepsilon_{i}^{2}\eta_{i}^{-1}\right)}^{-1}\left({\left(\alpha_{i}WB_{i}\left(x\right)\right)}^{2}-{\left(\omega_{i}^{sp}\beta_{i}\pi_{p}\right)}^{2}\right)\;+WB_{i}\left(x\right)\sum_{j=1}^{N}\alpha_{j}{\left(-2\omega_{i}^{dis}-2\omega_{i}^{eh}\left(1-\beta_{i}\right)\varepsilon_{i}^{2}\eta_{i}^{-1}\right)}^{-1}\left(\alpha_{j}WB_{j}\left(x\right)+\omega_{j}^{sp}\beta_{j}\pi_{p}\right)$$

□

## 4. Numerical Simulations

In this section, a series of numerical simulation experiments have been done using a mathematical software named MATLAB, version R2016a, to show the optimal strategy’s change over time about each of the SUs’ resource leased through the differential game model formulated and solved above. In the next portion, we comprehensively analyze the differential game model open loop Nash equilibrium solution and feedback Nash equilibrium solution to SUs about the spectrum band resource leased. In the simulations, for both the open loop and feedback situation, three SUs are chosen to make the simulation environment, and to show the dynamic changing process of the system strategy based on the proposed differential game model. All the differential game model simulation parameters that are used in the experiment process are shown in Table 1, where we see that there are some parameters (discount rate r, the unit price $\pi_{p}$ that the PU appointed to lease their spectrum resource, the spectrum loss rate δ during the process of spectrum leasing) that are the same for the three users; on the one hand, to simplify the simulations, we consider some special scenarios.

**Figure 3:**
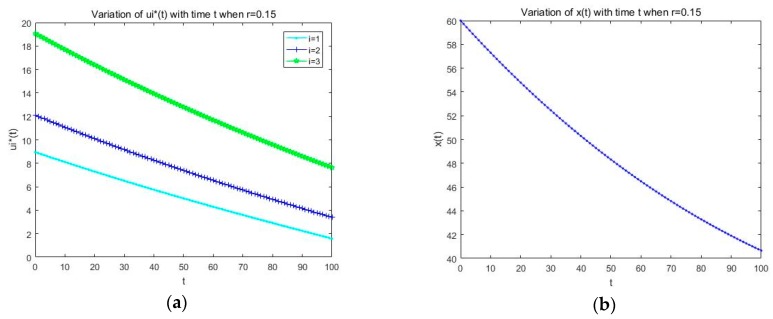
(**a**) Variation of $u_{i}^{*}\left(t\right)$ with time t for the open loop solution; (**b**) variation of x(t) with time t for the open loop solution.

**Figure 4:**
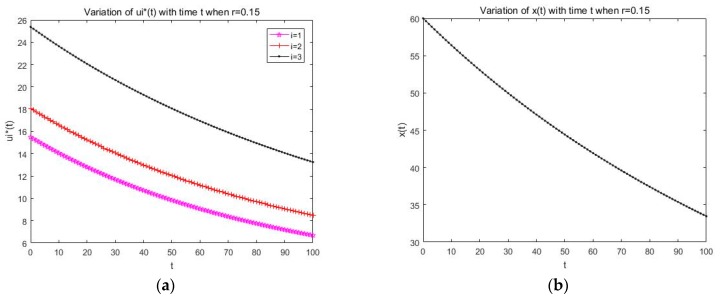
(**a**) Variation of $u_{i}^{*}\left(t\right)$ with time t for the feedback solution; (**b**) variation of x(t) with time t for the feedback solution.

Firstly, we analyze the open loop solution and feedback solution $u_{i}^{*}\left(t\right)$ of the differential game model that are formulated in this paper, and from Section 3, we have got the optimal resource allocation strategy expression of SU about the open loop Nash equilibrium and feedback Nash equilibrium. Through the optimal strategy expression and the parameters’ setting, we get the strategy simulation figure, and via the simulation in Figure 3a and Figure 4a, we see that the changing of the resource allocation strategy $u_{i}^{*}\left(t\right)$ over time is decreasing gradually and stabilizing to a certain value in both the open loop and feedback solution simulation figure, which satisfies the actual fact that we know $u_{i}^{*}\left(t\right)$ represents the spectrum resource that SU *i* leased from the PU at t instantaneous, and in the information transmission stage, the leased spectrum and the harvested energy are used by the SUs to complete certain information transmission tasks; as time goes by, some information transmission tasks may tend to be finished or stable, which results in that fewer spectrum resources may be needed with time than the previous moment, so the spectrum resource that needs to be leased from the PU may decrease gradually, and with transmission tasks finished or tending toward a stable state, some permanent spectrum resource may just be needed to maintain the message transmission status, so the curve trend is stable near to a certain value.

**Figure 5:**
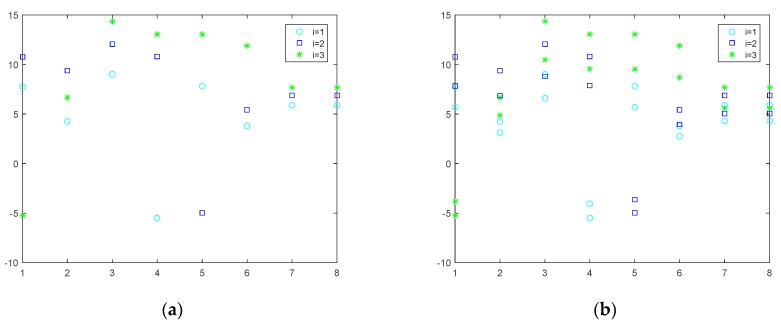
(**a**) Variation of $u_{i}^{*}\left(t\right)$ for the feedback solution under infinite horizon at *t* = 10; (**b**) variation of $u_{i}^{*}\left(t\right)$ for the feedback solution under infinite horizon at the end of the game.

Secondly, we analyze the variation of x(t) over time under the optimal resource allocation strategy $u_{i}^{*}\left(t\right)$ in both the open loop and feedback solution situation; from Formula (4) and same as with the analysis description of $u_{i}^{*}\left(t\right)$ in the above, we know *x*(*t*) represents the spectrum resource capacity that the PU wants to rent externally at t instantaneous and the variations of *x*(*t*) are relevant to not only the optimal strategy $u_{i}^{*}\left(t\right)$ but also *x*(*t*) itself. Here, through Formula (4), the optimal strategy $u_{i}^{*}\left(t\right)$, and the parameters’ setting, we get the changing simulation figure about x(t), Figure 3b that represents the change of x(t) with time under an open loop solution, and Figure 4b that represents the change of x(t) with time under a feedback solution situation. In addition, from Figure 3b and Figure 4b, we see that the trend of x(t) over time gradually decreases and stabilizes to a certain value, which satisfies the actual fact that, on the one side, with the information transmission tasks near to being finished, the resource of the spectrum band that the SU wants to lease from the PU may be reduces, which result in the consequence that the resource the PU wants to rent outside may be depressed to achieve the maximum of resource utilization. On the other side of the shield, long-term rental resources may cause the reduction of the spectrum used by the PU, which may bring about the reduction of its work efficiency to the PU; therefore, the spectrum that the PU rents externally may decrease over time. The optimal solutions in the feedback situation under an infinite horizon are also analyzed, which are given in Figure 5. Based on the results given in Figure 5, we can find that the system state will be changed with the time varying, and the optimal solutions would be changed, and there will be more optimal solutions for the users to choose.

## 5. Conclusions

In this article, the secondary users that have the energy harvest function can achieve the information transmission task via its stored energy and the spectrum resource leased from the PU. The act of leasing a spectrum resource brings about a cost increase for SUs. To minimize the cost, firstly, we formulated a differential game model to represent it mathematically and figuratively. Secondly, by solving the game model, the open loop Nash equilibrium solutions and the feedback Nash equilibrium solutions were obtained, which illustrates the fact that optimal resource allocation strategies for SUs exist. Finally, a certain number of numerical simulations was done to verify the correctness of the differential game model.

## Figures and Tables

**Table 1:** Simulation parameters setting in the differential game model.

Parameters	i	$\omega_{i}^{dis}$	$\omega_{i}^{eh}$	$\omega_{i}^{sp}$	$\beta_{i}$	$\varepsilon_{i}$	$\eta_{i}$	r	$\pi_{p}$	δ	$\alpha_{i}$	$\Phi_{i}$	T
Value	1	0.4	0.8	0.1	0.1	0.3	0.028	0.15	0.2	−0.15	−0.6	0.1	100
Value	2	0.5	0.4	0.3	0.4	0.5	0.03	0.15	0.2	−0.15	−0.5	0.6	100
Value	3	0.6	0.3	0.6	0.7	0.7	0.034	0.15	0.2	−0.15	−0.4	0.8	100
